# The Biomechanical Effect of Different Denture Base Materials on the Articular Disc in Complete Denture Wearers: A Finite Element Analysis

**DOI:** 10.3889/oamjms.2015.074

**Published:** 2015-07-13

**Authors:** Mohamed M. El-Zawahry, Ahmed A. El-Ragi, Mohamed I. El-Anwar, Eman M. Ibraheem

**Affiliations:** 1 *Prosthodontics Department, National Research Centre, Giza, Egypt*; 2 *Civil Engineering Department, Faculty of Engineering, Fayoum University, Egypt*; 3 *Department of Mechanical Engineering, National Research Centre, Egypt*

**Keywords:** TMJ Articular disc, stresses, Finite element analysis, Denture base material

## Abstract

**AIM:**

The objective of the present study was to evaluate the effect of different denture base materials on the stress distribution in TMJ articular disc (AD) in complete denture wearers.

**MATERIAL AND METHODS:**

A three dimensional Finite Element (FEA) models of an individual temporomandibular joint (TMJ) was built on the basis CT scan. The FEA model consisted of four parts: the condyle, the articular disc, the denture base, and the articular eminence skull. Acrylic resin and chrome-cobalt denture base materials were studied. Static loading of 300N was vertically applied to the central fossa of the mandibular second premolar. Stress and strain were calculated to characterize the stress/strain patterns in the disc.

**RESULTS:**

The maximum tensile stresses were observed in the anterior and posterior bands of (AD) on load application with the two denture base materials. The superior boundaries of the glenoid fossa showed lower stress than those on the inferior boundaries facing the condyle.

**CONCLUSIONS:**

Within the limitations of the present study it may be concluded that: The denture base material may have an effect in stress-strain pattern in TMJ articular disc. The stiffer denture base material, the better the distribution of the load to the underling mandibular supporting structures & reducing stresses induced in the articular disc.

## Introduction

The temporomandibular joint (TMJ), a load-bearing organ in the human body, contains an articular disc located between the glenoid fossa and the condyle that, during mandibular movements, plays an important role as a stress absorber during mouth function, resulting in stress reduction and redistribution within the joint [1].

The articular disc is taking important role in absorbing the load of the joint in function. Excessive load on the joint my lead to degeneration of the articular disc and surrounding structures, resulting of a pathological condition.

The group of ‘temporomandibular disorders’ (TMD) comprises a number of related clinical problems involving pain and dysfunction of the masticatory system, the temporomandibular joint and its associated structures. The main cause of TMD has not yet been established, although functional overloading is considered to be a major etiological factor [1].

Stress distribution in the TMJ is hard to be measured experimentally, thus it is poorly understood. However, finite element (FE) analysis is a promising research tool for evaluating dental biomechanics [2]. It can be used to analyze stress distribution patterns in the TMJ tissues after application of force or deformation. Two-dimensional (2D) and three-dimensional (3D) FE models have been used to simulate the in vivo biomechanics of the human TMJ [3].

Most previous studies focused on clenching behaviors, since maximum TMJ loading occurs during forceful clenching or aggressive episodes [4]. However, the TMJ is also sub-maximally loaded during many other activities, such as drinking, screaming, biting, and masticatory opening and closing [5]. The condylar movement during these various mandibular movements, especially jaw opening, produces remarkable ranges of disc mobility.

There would be varying opinions on the prevalence of TMD signs in dentate population. TMD appear to be almost as prevalent in complete dentures (CD) wearer as in dentate individuals, varying from 15 to 25%others reported that CD wearers were found to have a higher prevalence of TMD symptoms than the normal population with natural dentition [6].

The effect of denture condition on TMDs is controversial. One study found no statistically significant correlations between signs and symptoms of TMDs and denture retention, stability, occlusal disturbances, freeway space, age of present denture or the number of sets of dentures. However, some studies have shown that denture wearers have a higher prevalence of TMD symptoms compared to the normal population or to those who still have natural teeth, and that the incidence and intensity of TMDs are higher in subjects with greater tooth loss in the supporting zones [7, 8].

A complete denture is considered inadequate when it is unstable, present’s lack of retention, or when there is loss of vertical dimension to any extent, resulting from either incorrect manufacturing or wear of artificial teeth [9].

The use of inadequate complete dentures is considered as a reason for the development of oral lesions, such as denture stomatitis, angular cheilitis, traumatic ulceration and inflammatory hyperplasia [10]. It may also contribute to the development of TMDs.

On the contrary the immediate result of centric prematurity is displacement of the denture which acts as a buffer to the Temporomandibular Joint from dysfunction.

Several studies have found no correlations between certain characteristics of dentures (denture retention, stability, occlusal errors, freeway space, age of present denture, or number of sets of dentures and the presence or severity of TMD signs and symptoms. [11]. In addition, loss of vertical occlusal height on its own may not be responsible for the TMD occasionally seen in CD wearers with reduced vertical height [9].

The aim of this study was to evaluate the stresses induced in the TMJ articular disc on using different denture base materials in complete denture wearers.

## Material and Methods

A three dimensional finite element model of TMJ based on CT scan images was specially constructed, using sequential CT images at 0.5 mm cut thickness. The CT images were saved in Dicom format. Starting from the CT images, an intermediate image processing program Mimics^1^ Vr. 10.01 transferred the huge amount of gray scaled colored points on the CT images acquired in the successive images into cloud of points in Cartesian coordinates.

**Figure 1:**
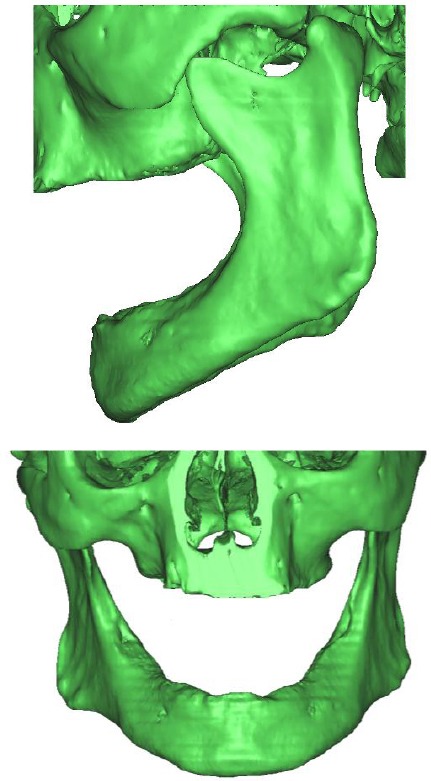
*Three dimensional geometric model obtained from CT images*.

**Figure 2:**
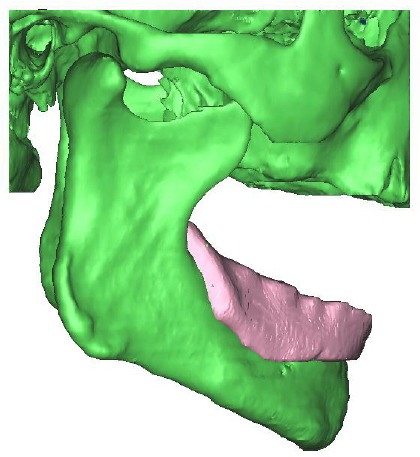
*Three dimensional geometric model with denture*.

While, each range of a color (brightness and intensity indicating density of certain material or tissue type) and its associated points were gathered into set of mesh elements representing part of the model [9]. The program had selected portions of the skull using the “Threshold Operation”, which selects a range of brightness values marking only bony tissue, to create each model part mask. However, further thresholding removes all other tissues that are not included in this study. The gaps between successive images were subsequently filled in using the “Fill Operation”, which results in a solid mask with all the bony tissues as presented in Figures 1 and 2.

The finite element model consists of four main parts: condyle, articular disc, denture base, and articular eminence (skull). Element Solid-l87 (tetrahedral structural solid element), was used in meshing the model on ANSYS^2^ finite element software version 12.0.

Such 3D element is well suited to modeling irregular meshes such as those produced from various image processing and CAD/CAM systems. This element is defined by 10 nodes having three degrees of freedom at each node (translations in the nodal x, y, and z directions) [10].

**Figure 3:**
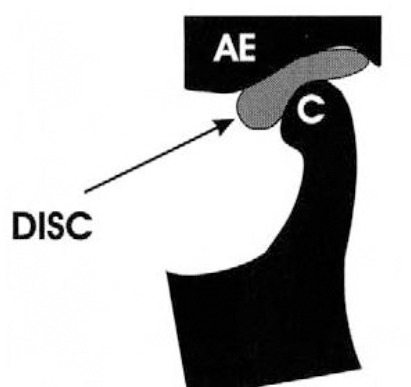
*Schematic diagram showing the articular eminence (AE) of the temporal bone, mandibular condyle (C) and the disc (indicated with an arrow) [11]*.

**Figure 4:**
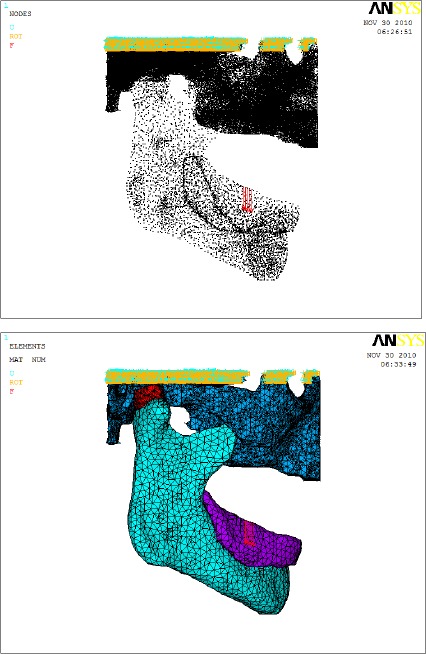
*Meshed model with denture (colors indicate different materials)*.

Upon reconstruction of the condyle and articular eminence, a disc was added between them with a uniform thickness of 2 mm, as illustrated in Figures 3 and 4, where each color represent different material or tissue, and red arrows indicate the applied load.

Linear elastic material properties were assigned to each part of the model taken from the literature as listed in Table 1. Two material for denture base were tested, one for lowest usable value and one for highest usable value of Young’s modulus as 300, and 200,000 MPa respectively. The boundary conditions were directly written to the ANASYS command by custom developed software, by fixing the top areas of the model. The solid modeling and Finite Element Analysis (Linear static analysis) were performed on a personal computer Intel Pentium IV, processor 2.8 GHz, 1.0GB RAM.

**Table 1:** Material properties [Young’s modulus (E) and Poisson’s ratio (γ)

	Young’s modulus	Poisson’s ratio
Acrylic resin (denture base)	3000 MPa	0.30
Metallic alloy (denture base)	200,000 MPa	0.30
Bone	11,500 MPa (average)	0.33
Articular disc	6.82 (average)	0.40

## Results

Four graphical result representations had described the mechanical behavior of the TMJ articulating disc (AD); vertical (U_Z_), horizontal (U_X_) displacements in mm, maximum tensile (S_1_) and maximum compressive (S_3_) stresses in MPa.

**Figure 5:**
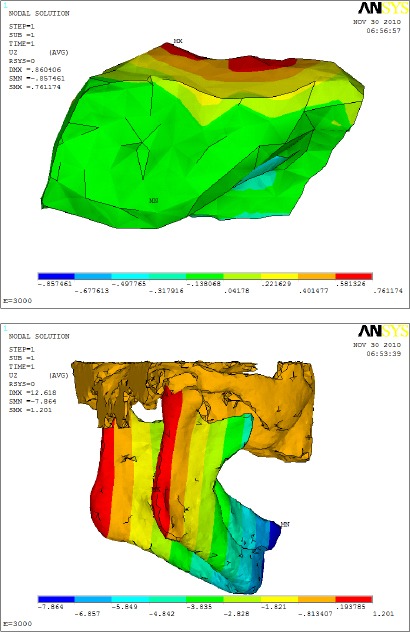
*Vertical displacement (U_z_) distribution with acrylic resin denture base material*.

**Figure 6:**
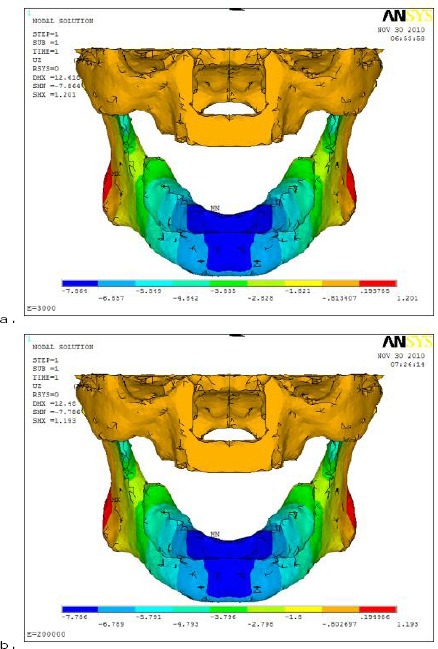
*comparison between vertical displacement (U_z_) distributions on mandible with using different denture base materials (a- Acrylic resin and b- metallic alloy)*.

As shown in Figures 5 and 6: it was found that the vertical displacement (U_z_) of the mandible had not been affected by the type of the denture base material, (-7.864 mm for the acrylic base material versus -7.786 mm for Metallic denture base material).

**Figure 7:**
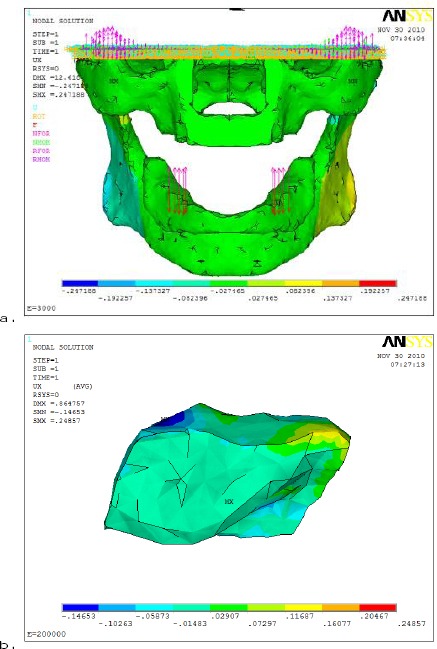
*comparison between horizontal displacement (U_x_) distributions with different denture base materials (a- Acrylic resin and b- metallic alloy) on mandible and AD*.

On the other hand, the horizontal displacement (U_x_) results in Figure 7, had shown that the opening of the rear part of the mandible (summation of absolute horizontal displacement values in two different directions).

The use of different denture base materials did not affect the horizontal displacement magnitude noticeably.

Figures 5 and 6 revealed that the inter-condylar distance had increased with the reduction of the denture base material Young’s modulus of elasticity from 200,000 (Metal Base) to 3,000 MPa (Acrylic Base).

**Figure 8:**
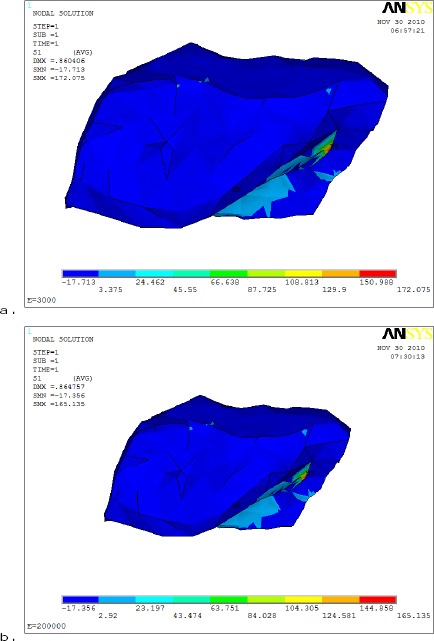
*comparison between maximum tensile stress (S_1_) distributions on articular disc using different denture base materials (a- Acrylic resin and b- metallic base)*.

Figure 8 showed that the maximum tensile stresses (S_1_) on the articular disc was172.075 MPa and 165.135 MPa with Acrylic resin &metallic denture base materials respectively. i.e the maximum tensile stresses in the AD are decreased by about 4.2% with increasing the rigidity of the denture base.

**Figure 9:**
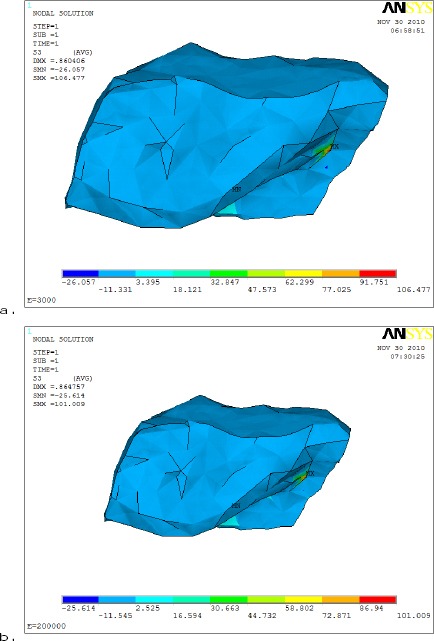
*Comparison between maximum compressive stress (S_3_) distributions on articular disc using different denture materials (a- Acrylic resin and b- metallic base)*.

Figure 9 showed the maximum compressive stress (S_3_) distribution was in the intermediate zone of the AD. The maximum compressive stress (S_3_) was 26.057 MPa to 25.614 MPa with (Acrylic base& Metal base respectively in the articular disc area, i.e. the maximum compressive stress was 1.73% larger in acrylic than the metallic denture base.

The results of the present study revealed that the more rigid the denture base material, the better the distribution of the load to TMJ articular disc & mandibular arch.

## Discussion

The TMJ may be one of the most heavily load-bearing joint in the human body, so its biomechanical balance has great significance to its function [3]. The articular disc in the TMJ acts as a stress cushion during TMJ activity, so analysis of stress distribution throughout this disc during function is of great importance.

Understanding the nature of strain and stress distribution in TMJ disorders is essential for better diagnosis and treatment of stomatognathic diseases and reconstruction of masticatory function [2].

Unfortunately, the stresses cannot be measured directly in a non-destructive way. The number of direct studies on the masticatory system is limited, because its structures are difficult to reach and the applications of experimental devices, such as strain gauges, inside the structure introduce damage to its tissues, which interfere with normal function and influence their mechanical behaviour [2, 6].

FE models of the TMJ were developed to investigate stress and reaction forces within the TMJ during clenching [12-14] and chewing.

The adequacy of the FE computational model to the real system depends on the correctness of representation of the geometry and material properties of the modeled object, the type and number of elements and the boundary conditions imposed on the model [4].

General point is that the precision of finite element calculations increases as highly refined meshes of the model parts are used. On the other hand, it is well known that highly refined meshes are necessary only at zones where high stress gradients are expected and at zones of complex and highly curved geometrical shapes of the model [15].

The jaw dimensions including the length, arch curvature, height, and cross sectional dimensions of both cortical and cancellous bone were obtained from a CT of the patient to transfer them to sketches in solid works program [15].

The nature of contact between the denture base and the surrounding bone has been referred to as slip or no penetration contact In order to simplify the computations of stress analysis. This simplification was performed because the main goal of this study was to evaluate the possible effect of using different denture bases on the stress distributions in the temporomandibular joint discs [16].

The CT cut sections were approximately recorded to overcome the problem of compact and cancellous bone distribution in the mandibular model. Moreover, to obtain the correct representation of the geometry of the model the minimal slice thickness possible to be registered by the CT device was chosen as recommended by [15, 17].

The mechanical behavior of the TMJ disc, when investigated experimentally in humans [18] was found to be nonlinear, anisotropic and time-dependent, and varied between different regions of the disc. However, in most previous studies [1, 19] the material properties of the disc were considered as entirely homogeneous.

Static load was utilized in this study which is inconsistent with the dynamic nature of loading. As many researches had proofed that simulation of dynamic loading is quite difficult and may require sophisticated equipment and may produce a huge of inter-related results which can’t be explained [19].

The vertical load was applied to the areas of central fossae of the premolar teeth to assure transmission of vertical occlusal stresses to the underlying supporting structures, reduce the lateral and oblique forces delivered to the underlying tissues [20].

The mechanical behavior of the TMJ disc, when investigated experimentally in humans, most previous studies focused on clenching behaviors, since maximum TMJ loading occurs during forceful clenching or aggressive episodes [4].

However, the TMJ is also sub-maximally loaded during many other activities, such as drinking, screaming, biting, and masticatory opening and closing. The condylar movement during these various mandibular movements, especially jaw opening, produces remarkable ranges of disc mobility [5].

The present study was conducted to evaluate the effect of denture base material on the stresses generated in TMJ articular disc. Acrylic resin and Chrome Cobalt denture base materials were utilized as they are the most commonly used denture base materials under functional masticatory activity.

The results of the present study revealed that: The highest tensile stresses were observed in the anterior and posterior bands of the articular disc during joint loading in the two studied models; this finding may be due to transmission of larger percentage of occlusal loads to those areas during joint loading or biting as the disc may be presented into close contact with the condylar head.

This finding may agree with [19] who reported that during jaw opening, the intermediate zone bears mainly compressive stress. However, the anterior and posterior bands bear mainly tensile stresses.

However, the superior boundaries of the articular disc in contact with the glenoid fossa exhibited low stresses than those on the inferior boundaries facing the condyle during loading; as the major part of the transmitted occlusal forces had been already absorbed by the condyles and the undersurface of the discs [15].

The results had revealed that the metallic denture base had transmitted less stresses to TMJ articular disc. Those results agree with [21] who reported that whenever the rigidity of denture base is high, the strain of dentures decreases, the stress of the residual mucous membrane will evenly become distributed, and the distance of the balancing side from the denture border to the residual mucous membrane at the time of unilateral balanced position will decrease.

The low tensile stress values observed with the metallic denture base may be due to the higher rigidity of Co-Ch alloy which may limit the mandibular flexure and consequently decreasing the stresses delivered to the articular disc [21].

The relatively higher tensile stresses noticed in the articular disc with acrylic resin denture base in comparison to Co-Ch may be due the less rigidity of acrylic resin that induce more flexure of the mandibular arch. Moreover, the inter-condylar distance may be increased with the mandibular flexure leading to higher tensile stresses induced in the articular disc.

Within the limitations of the present study it may be concluded that:

- The denture base material may have a minor effect on stress-strain pattern in TMJ articular disc.
- The stiffer the denture base material, the better the distribution of the load to the underling mandibular supporting structures & reducing stresses induced in the articular disc.
- Generally, the stress and strain remained at a reasonable level during normal jaw movements during mastication, indicating that the disc experiences no injury during functional activities in a healthy joint.

## Footnotes

1 Materialise, Technologielaan 15, 3000 Leuven, Belgium

2 ANSYS Inc., Canonsburg, PA, USA.
